# Is a picture worth the same emotions everywhere? Validation of images from the Nencki affective picture system in Malaysia

**DOI:** 10.1007/s44192-024-00116-y

**Published:** 2024-12-02

**Authors:** Elizaveta Berezina, Ai-Suan Lee, Colin Mathew Hugues D. Gill, Jie Yun Chua

**Affiliations:** 1 Department of Psychology, Sunway University, Bandar Sunway, Malaysia; 2 Universal College Bangladesh, Dhaka, Bangladesh; 3 Department of Economics, University of Warwick, Coventry, UK

**Keywords:** Nencki affective picture system (NAPS), Emotion induction, Affective ratings, Affective image set, Cross-cultural differences

## Abstract

Several validated image sets, such as NAPS, IAPS, GAPED, and OASIS, have been developed to elicit affective states. However, these image sets were primarily validated on Western populations within European and American contexts, and none have been fully validated in a Southeast Asian sample, where emotional restraint may also be valued similarly to the East Asian contexts. This study aimed to validate and provide norms for the Nencki Affective Picture System (NAPS; Marchewka et al., 2014) within a Malaysian sample. Subsets from the 1356 NAPS images consisting of five image categories (faces, people, objects, landscapes, animals) were presented sequentially to 409 Malaysian adults aged 18 and above, who rated images for valence, arousal and approach/avoidance on a 9-point Likert scale. Valence, arousal and approach/avoidance norms were compared against the original European sample. Malaysian men and women rated images with lower valence and motivation than Europeans, but Malaysian men showed higher arousal ratings compared to European men, while Malaysian women exhibited the opposite pattern, with lower arousal ratings than European women. A linear regression was found instead of a classic ‘boomerang’ shaped quadratic regression previously observed in Western samples, suggesting that emotional suppression may be at play, in line with social norms. The Malaysian normative ratings will be freely available to all researchers.

**Supplementary Information:**

The online version contains supplementary material available at 10.1007/s44192-024-00116-y.

## Introduction

Emotions play a vital role in human social interactions and well-being. Emotional experiences involve multifaceted processes such as evaluation, regulation, and expression, arising from an interplay of valence (the perceived positiveness or negativeness [1], arousal (the subjective state of activation [2], and motivational states (the approach/avoidance responses that scale with reward/threat value [3, 4]. These core affective dimensions interact to shape discrete emotional experiences [5, 6].

The theoretical background that justifies the choice of the three dimensions of affective states lies in two foundational theories, namely the circumplex model of affect [7–9] and its expanded version with a third added dimension [4]. The initial circumplex model of affect, also known as the valence-arousal model, organises emotions on a circular graph with two dimensions: (1) displeasure/pleasure (valence) and (2) deactivation/activation (low vs. high arousal). Emotions exist on a continuum based on the combination of valence and arousal (e.g., anger has low valence and high arousal), with core affect placed at the centre of the circle, representing a neutral state [9]. The addition of a third dimension to the circumplex model was proposed [4]: approach-avoidance motivation that provides a more comprehensive framework for understanding emotions. The approach-avoidance motivation presented as two general activation systems: Positive Activation is associated with the goal-directed behaviour, high energy and pleasantness, whereas Negative Activation is linked to more negative emotions and withdrawal behaviour. This dimension helps to explain why some emotions with similar valence and arousal (e.g. fear and anger) can lead to different behavioural outcomes. Therefore, these dimensions provide a robust foundation for investigating how individuals perceive and respond to emotional stimuli, allowing for better distinguish affective responses. Research into emotions requires reliable elicitation of affective states within experimental contexts. Validated stimulus sets comprised of images standardized on emotional impact provide crucial tools enabling precise induction of target affective states [10, 11]. Systematic stimulus validation using participant ratings of valence and arousal facilitates reliable selection of stimuli to provoke discrete emotions [12]. Standardized image sets containing sufficiently large samples classified across affective dimensions are vital for experimental control and measurement in emotion research, indexing mechanisms spanning attention and memory to decision making and behaviour [13–15].

Recent research has shown that the ways in which participants respond to visual stimuli is closely linked to the affective processes underlying moods and emotions [16–19]. Visual stimuli can induce changes in affective states [20–22] and the ways in which participants process and respond to images has been shown to be associated with affective disorders [23]. Although the precise nature of the links between stimulus images, response associated changes in psychophysical biomarkers, and affective processes is not yet fully understood [24], standardized images may provide a viable and quick means to assess affective processes and disorders in those processes [25–28].

### Emotional processes

The terms emotion, mood and affect are often used interchangeably and without clear definitions, which can lead to confusion when comparing different research methods and results [29]. Emotions are a complex pattern of changes that embody feelings, cognitive processes, physiological arousal and behavioural reactions. The technical definition of emotions largely depends on the theoretical framework chosen, but in general, emotions can be summarised as short-lived reactions to an event or stimuli [30]. In comparison, moods are low intensity, subjective feelings, relatively long in duration [31]. Moods are a fundamental component of everyday life that can have a significant influence on an individual’s well-being, psychological health and cognitive functioning [32]. Although moods and emotions may vary in specific ways, they both interact and influence one another; emotions can turn into moods, while moods can alter an emotional response to an event [33]. Everyday mood fluctuations influence executive functioning and processes such as attention, working memory, problem-solving, creativity, planning and behavioural control [34]. A positive or negative mood can thus determine how information is processed, interpreted and evaluated [35, 36], influencing an individual’s pre-conceptions, perspective, attitude, judgements, decision-making and behaviour [32, 37]. Sustained disturbances in mood, whether arising from positive or negative emotional states, may lead to a wide range of associated disorders that can adversely impact mental health over the lifespan [38].

As emotions are universal, it comes as no surprise that associated mood disorders are among the most common and detrimental illnesses that have a significant impact on quality of life and personal economics [39–41]. Emotional and cognitive disturbances contribute to the clinical symptoms of these disorders and decrease individuals’ ability to regulate the processes that lead to mood formation [42]. Therefore, interventions that can improve emotional self-regulation and promote positive moods could be advantageous in the development of prevention and treatment strategies [43], particularly if the interventions are simple and non-invasive, such as those that involve images [44–46].

### Cultural differences in affective processing

Several influential affective image sets exist, notably the International Affective Picture System (IAPS; [47]), the Geneva Affective Picture Database (GAPED; [48]), the Open Affective Standardized Image Set (OASIS; [49]), the Categorized Affective Pictures Database (CAP-D; [50]), the DIsgust-RelaTed-Images (DIRTI) database [51] and the Nencki Affective Picture System (NAPS; [52, 53]. However, these image sets were primarily validated on Western populations within European and American contexts. Cross-cultural research demonstrates that while some emotional processes are universal [54], cultural variables including display rules and values shape emotional regulation and expressions [55]. As NAPS standardisation data from the West may not be applicable in Asia [56] this study addresses a major research gap by standardizing the NAPS images for use with Malaysian participants.

A substantial amount of evidence suggests that Asian participants may rate affective images less intensely than Americans, possibly reflecting more collectivist cultural values [58–60]. Response patterns also differ across Asian cultures; for instance, boomerang-like arousal ratings observed in Europeans (where very positive and negative valence was associated with higher arousal [52]) were absent in older Chinese adults [56] and showed a less-curved boomerang shape in an Indian sample [61]. Furthermore, differences emerged between Iranians and Germans in their arousal and dominance patterns [62].

Furthermore, there are notable cultural value differences between various Asian societies (e.g. China, Japan, India etc.) and Southeast Asia (e.g. Malaysia, Indonesia) based on Hofstede’s cultural dimensions [63, 64]. Southeast Asian countries tend to rank lower on Confucian-driven values of long-term orientation and is higher on dimensions like indulgence versus restraint than East Asians [65]. This suggests emotion norms developed in East Asian samples [56, 66] may not correspond to Southeast Asian contexts. Malaysia in particular has synthesis of indigenous, Malay, Chinese, British and Indian influence on socio-emotional norms [67] alongside widespread exposure to Western movies and media [68]. This combination of Eastern values and extensive Western media familiarity suggests that differences in valence, arousal and approach/avoidance patterns may exist in Southeast Asian samples.

### Gender differences in affective processes

Research has shown that males and females may experience different level of arousal and emotional response to the same visual stimuli [69–71]. The original study validating NAPS found that the correlation between arousal and valence is stronger in women than in men [52]. Similarly, Lithari et al. [69] demonstrated that that women exhibit greater event-related potential amplitudes in response to unpleasant and highly arousing stimuli. In a study on a Portuguese sample, males and females reacted differently to emotional images in terms of pleasantness and arousal [70]. However, some studies present contradictive findings regarding the gender differences. For example, Mikkelsen et al. [72] found that women rated sad and disgusting images as more arousing than man, while no gender differences were observed in valence ratings for the overall set, sad and happy images. Another study found no significant differences between male and female smokers in their neurophysiological responses to cigarette-related images or other emotional stimuli [73]. Overall, the findings suggest that women may have a heightened sensitivity to emotional stimuli, particularly unpleasant images, however, it's important to note that not all studies find consistent gender differences in responses to emotional stimuli, therefore it should be explored further.

### Current study

Much recent research on affective processes has used the Nencki Affective Picture System (NAPS) [52], a set of 1356 images which have been standardized based on the fundamental dimensions of Valence, Arousal and Approach/Avoidance which are hypothesised to underly affective processes [74, 75]. These images have been standardized for use across several western populations (e.g. [26, 76]), but have not been standardized for use in Malaysia. It is particularly important that such images are standardized in target populations because there is evidence that people from Western and Eastern cultures respond to images differently and, therefore, that standardization data from the West may not be applicable in Asian contexts [57].

More specifically, there is still a lack of affective image sets that have been validated for a Malaysian sample. Thus far, only two studies have examined affective responses to visual stimuli among Malaysian participants. Abdul Rahman and Reza [77] provided Malaysian norms from a subset of 166 images from the International Affective Picture System (IAPS), while Nasser et al. [16] used a separate set of images (consisting of negative emotional cues and neutral cues) that were obtained from publicly-available Instagram accounts. However, neither study provided norms for the complete image set. In particular, the Nencki Affective Picture System (NAPS) has not been normed for a Malaysian sample. Validating the full NAPS image set with Malaysian participants in the current study would provide the first cultural adaptation of this tool for use in Malaysia, thus providing Malaysian—and potentially Southeast Asian—researchers with an alternative, validated image set to the widely-used IAPS to induce and assess emotional states using visual images.

Therefore, the aims of this study are (1) to identify culturally appropriate Nencki Affective Picture System (NAPS) visual stimuli for assessing affective processes in Malaysia; (2) to validate images from the NAPS for use with Malaysian participants and compare with the original European norms [52]. We hypothesised that there will be significant cultural differences in the valence, arousal, and approach/avoidance when individuals evaluate NAPS images. Additionally, (3) we predict gender differences in the affective ratings among Malaysian participants, as well as with the original European sample.

## Methods

### Study design

The study used a cross-sectional design to measure valence, arousal and approach/avoidance indices of 1356 images from the Nencki Affective Picture System (NAPS) database [52] in Malaysia. The protocol was approved by the University Research Ethics Committee in accordance with the Malaysian Code of Responsible Conduct in Research (2nd Edition) and the Belmont Report.

### Participants

An a priori power analysis using G*Power version 3.1.9.7 [78] was conducted to determine the minimum sample size required. To achieve 80% power for detecting a medium effect of *η*^*2*^ = 0.5, at a significance criterion of α = 0.05, the recommended sample size was *N* = 176. To ensure that each image was evaluated ≥ 5 times, the minimum sample size was increased. Therefore, the current sample of 409 participants (*M*_age_ = 21.20, *SD* = 2.97; 26.4% males, 64.10% females; 9.7% other or did not indicate their gender) was sufficient to test the hypotheses and evaluate all NAPS images. All participants were Malaysians who had normal or corrected-to-normal vision as well as normal colour vision; most were students or staff at the University who had professional working proficiency or higher in English.

Comparisons with the Western sample were based on Marchewka et al.’s [52] ratings obtained from a European sample (60% Polish, 40% other European nationalities; mean age = 23.9 years, *SD* = 3.4).

### Measurements and materials

#### NAPS image stimuli

The stimuli used in the study were obtained from the Nencki Affective Picture System (NAPS) database [52]. The NAPS database is composed of five broad categories, namely people, faces, animals, objects, and landscapes, encompassing positive, negative, neutral images, with low, neutral and high arousal, as well as approach/avoidance indices. An initial review of the 1356 NAPS images was conducted by a pool of Malaysian researchers to identify images that might be culturally insensitive for use in Malaysia (e.g. religious sensitivities); no images were excluded.

##### Rating/sorting

The image rating task was performed on Labvanced software ([79]; https://www.labvanced.com/). The Labvanced platform has been widely used in recent published studies (e.g. [80–82]), and has been shown to be particularly effective for the presentation of visual stimuli [83] and the capturing of affective process ratings [84].

To ensure that participants were not overwhelmed, the research team divided the 1356 images into 12 image sets, with each set consisting of 113 images. The Labvanced platform randomly presented each participant with 35 images. Thus, each participant viewed and rated randomly assigned 35 images, with no more than three images from the same category presented consecutively. Participants were asked to rate each image based on their first impression, but no time limit per image was given.

**Fig. 1:**
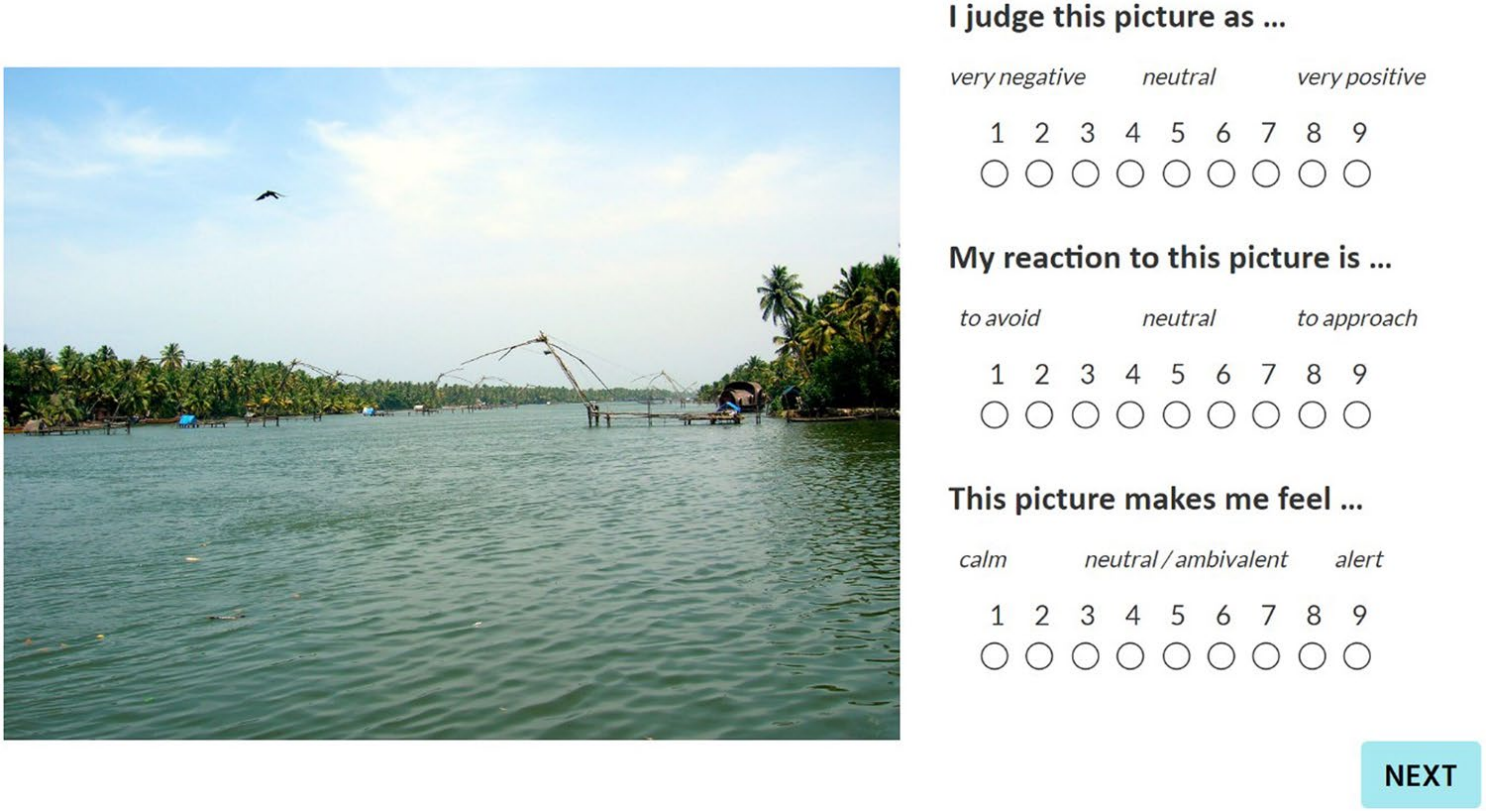
Screenshot of the rating task on the Labvanced experimental platform

All ratings (valence, arousal, and avoidance/approach) were measured using 9-item Likert scales. Modifications were made to the original rating scales in Marchewka et al. [52] to increase the clarity of the rating instructions for non-native English speakers. Items were rephrased from the original “You are judging this image as …" to “I judge this picture as …” (1 = very negative, 9 = very positive) on the valence scale and “Confronted with this image, you are feeling …” to “This picture makes me feel …” (1 = calm, 9 = alert) on the arousal scale. The avoidance/approach was measured using the following: “My reaction to this picture is… “(1 = to avoid; 9 = to approach) (see Fig. 1).

### Measurements

#### Positive and negative affect schedule (PANAS)

The 10-item Positive and Negative Affect scale (PANAS; [85]) consists of two subscales, 5 items each that measure Positive Affect (PA) and 5 items that measure Negative Affect (NA). Participants were asked to indicate to what extent they experienced the following feelings and emotions (e.g. upset, hostile) today using a 5-point scale (1 = very slightly or not at all; 5 = extremely) over the past week. The scale has a reasonably good internal consistency with Cronbach’s alpha 0.7 for both Negative and Positive Affect in the current study. Upon screening the sample, it was found that the mean fell within a moderate range of positive and negative affect (M_PA=_ 13.73, SD_PA_ = 4.78; M_NA_ = 10.56, SD_NA_ = 4.90). No participants were excluded due to their PANAS score.

#### DASS-8

The 8-item Depression, Anxiety and Stress Scale (DASS-8, [86]) is a shorter version of original DASS-42 and DASS-21 scales aimed at measuring depression, anxiety and stress. Participants were asked to indicate how much the statement applied to them over the past week (e.g. I felt that I was using a lot of nervous energy) using 4-items Likert scale (1 = never; 4 = almost always). This scale has good internal consistency with a Cronbach’s alpha of 0.86 in this study. In line with previous studies [86, 87], all eight items were summed. The sample was screened for extreme scores to ensure that overall DASS-8 scores were not skewed; the mean fell within a mild-to-moderate range of psychological distress (*M* = 16.67, *SD* = 4.31). No participants were excluded due to their DASS score.

### Procedure

Before the experimental session, participants were presented with an information sheet and consent form. After giving their informed consent, participants were asked demographic questions (age and gender), then screened for depression, anxiety and stress using the 10-item Positive and Negative Affect Scale (PANAS-10) and Depression Anxiety Stress Scales (DASS-8).

After completion of the questionnaires, participants were randomly assigned to a link to one of 12 image sets, each consisting of 113 images per set. Out of these 113 images, 35 images were randomly presented to each participant on the screen via the Labvanced platform. The images were rated using valence, approach/avoidance, and arousal scales from the original study on a 9-point Likert scale. Participants were asked to rate the images based on their first impression; however, no time limit was given for the rating task. After the image rating task, participants were presented with a debriefing statement and thanked for their participation. Participants received no compensation for this study.

### Data analysis

Data were analysed using IBM SPSS version 27 software. Three two-way ANOVAs were conducted to examine if cross-cultural and cross-gender differences in valence, arousal and approach/avoidance ratings towards the images. A quadratic regression was conducted to explore the valence-arousal relationship in the Malaysian and European samples.

## Results

The research team ensured that each of the 1,356 images was rated ≥ 5 times by participants. As different participants rated different images across 12 image sets, ratings for all 1,356 images were aggregated by gender and category (animals, landscapes, faces, people, objects), in line with Marchewka et al. [52]; in other words, each image was treated as a “participant” for the analysis.

### Comparisons between nationality and gender

In order to test the hypotheses, three 2 × 2 (nationality: ratings by Malaysians vs. Europeans and gender: ratings by men vs. women) repeated-measures ANOVAs were conducted separately for Valence, Arousal, and Motivation (approach/avoidance). Mauchly’s Tests of Sphericity revealed that the data met assumptions of sphericity (all *p*’s > 0.05).

#### Valence

The main effect of nationality was significant, *F*(1, 1187) = 248.76, *p* < 0.001, *η*_*p*_^*2*^ = 0.17. Pairwise comparisons showed that Malaysian participants (*EMM* = *4.97, SE* = 0.05) provided significantly lower valence ratings than the European sample (*EMM* = 5.40, *SE* = 0.05; mean difference = 0.43, *p* < 0.001); *t*(1355) = 17.57, *p* < 0.001, Cohen’s *d* = 0.48.

The main effect of gender was marginally significant, *F*(1, 1187) = 3.73, *p* = *0.05, η*_*p*_^*2*^ = 0.003. Pairwise comparisons showed that images were rated marginally higher in valence by male (*EMM* (estimated marginal means) = 5.21; *SE* = *0.05*) than female participants (*EMM* = 5.16, *SE* = 0.05; mean difference = 0.05, *p* = 0.05); *t*(1187) = 1.93, *p* = 0.054, Cohen’s *d* = 0.06. The interaction effect between nationality and gender was marginally significant, *F*(1, 1187) = 3.97, *p* = 0.05, *η*_*p*_^*2*^ = 0.003.

After applying Bonferroni correction, only the main effect of nationality remains statistically significant. Malaysian men and women provided lower valence ratings than those from the European samples (males: *t*(1194) = 12.37,* p* < 0.001, *d* = 0.36; females: *t*(1348) = 13.60, *p* < 0.001, *d* = 0.37). However, no gender differences were found within the Malaysian sample, *t*(1187) = 0.04, *p* = 0.97, *d* = 0.001, suggesting that Malaysian men and women provided similar valence ratings.

#### Arousal

The main effect of nationality was significant, *F*(1, 1187) = 13.38, *p* < 0.001, *η*_*p*_^*2*^ = 0.01. Pairwise comparisons showed that arousal ratings by Malaysian participants (*EMM* (estimated marginal means) = 5.20, *SE* = 0.04) were higher than ratings by European participants (*EMM* = 5.10, *SE* = 0.03; mean difference = 0.11, *p* < 0.001); *t*(1355) = 4.08, *p* < 0.001, *d* = 0.11. However, the main effect for gender was non-significant *F*(1, 1187) = 1.39, *p* = 0.24, *η*_*p*_^*2*^ = 0.001, as was the interaction between nationality and gender *F*(1, 1187) = 0.35, *p* = 0.56, *η*_*p*_^*2*^ < 0.001. After applying the Bonferroni correction, the main effect of nationality remains statistically significant.

#### Motivation (approach/avoidance)

The main effect of nationality was significant, *F*(1, 1187) = 216.04, *p* < 0.001,* η*_*p*_^*2*^ = 0.15. Pairwise comparisons showed that images were rated higher in motivation by the original European sample (*EMM* (estimated marginal means) = 5.38, *SE* = 0.04) than by Malaysians (*EMM* = 4.93, *SE* = 0.04; mean difference = 0.44, *p* < 0.001); *t*(1355) = 16.60, *p* < 0.001, *d* = 0.45.

Additionally, the main effect of gender was significant, *F*(1, 1187) = 5.03, *p* = 0.03, *η*_*p*_^*2*^ = 0.004. Pairwise comparisons indicated marginally higher motivation means for males (*EMM* = 5.18, *SE* = 0.04) compared to females (*EMM* = 5.13, *SE* = 0.05; mean difference = 0.05, *p* = 0.03); *t*(1187) = 2.24, *p* = 0.03, *d* = 0.07.

A significant interaction between nationality and gender was also found *F*(1, 1187) = 4.77, *p* = 0.03, *η*_*p*_^*2*^ = 0.004. Similar to the valence ratings, motivation ratings were significantly higher among European men and women compared to Malaysian men and women (men: *t*(1194) = 11.794, *p* < 0.001, *d* = 0.34; women: *t*(1348) = 12.657, *p* < 0.001, *d* = 0.35). No gender differences in approach/avoidance ratings were found in the Malaysian sample *t*(1187) = 0.10, *p* = 0.92, *d* = 0.006. After applying Bonferroni correction, only the main effect of nationality remains statistically significant.

#### Quadratic regression of the Valence vs. Arousal affective space

**Fig. 2:**
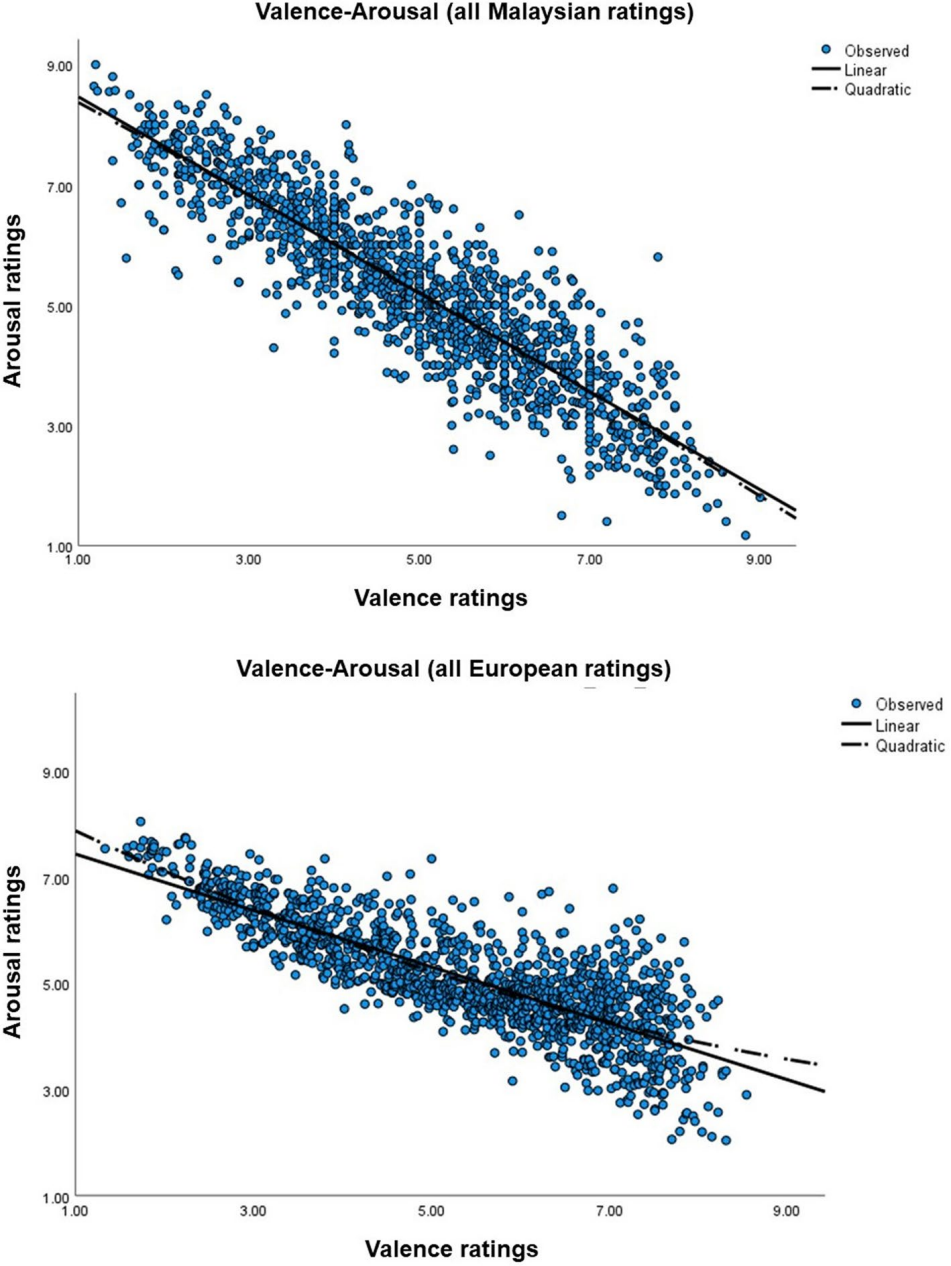
Quadratic function fitting arousal to the whole range of valence, for ratings of NAPS pictures by the Malaysian (upper) and original European samples (lower)

In line with previous cross-cultural validation studies (e.g. [52, 56, 62]), separate regression analyses for each sample were conducted to explore the relationships between valence and arousal for the Malaysian and original European sample by Marchewka et al. [52]. Unlike the original European sample however, no ‘boomerang’ shape was observed in the Malaysian sample, suggesting possible cultural differences. This was quantified by a non-significant quadradic model (*R*^*2*^ = 0.80, unstandardized *B* = −0.007, *p* = 0.23) in the Malaysian sample compared to a significant quadratic regression model (*R*^*2*^ = 0.80, unstandardized *B* = −0.82, *p* < 0.001) in the original European sample (see Fig. 2).

Although the above comparisons did not reveal significant gender differences in the Malaysian sample, prior research has indicated the existence of gender disparities in emotional regulation and expression within the Malaysian context [88, 89], which may still lead to variations in the valence-arousal quadratic space.

Prior to conducting separate quadratic analyses for Malaysian men and women, a multiple regression was conducted to examine whether valence, gender (dummy coded: 0 = male, 1 = female), and their interaction predicted arousal ratings. The regression model explained a significant proportion of variance in arousal ratings, *R*^*2*^ = 0.64, *F*(3, 2540) = 1499.60, *p* < 0.001. Valence significantly predicted arousal ratings (β = − 0.80, *SE* = 0.017,* p* < 0.001), while gender (β = 0.009, *SE* = 0.12,* p* = 0.81) and the interaction between valence and gender (β = 0.000, *SE* = 0.02, *p* = 1.00) were not significant predictors.

Nevertheless, to understand better where these differences may lie in the Malaysian sample, separate quadratic regression analyses were then conducted to understand the relationship between mean valence and mean arousal scores in Malaysian men and women.

##### Malaysian men

The linear regression model indicated that mean valence accounted for 56.8% of the variance in mean arousal, *F*(1, 1193) = 1569.26, *p* < 0.001, with higher mean valence associated with lower mean arousal. The quadratic regression model was non-significant (*R*^*2*^ = 0.001, *p* = 0.86), suggesting that the relationship between valence and arousal among Malaysian men was strictly linear.

##### Malaysian women

For Malaysian women, mean valence accounted for 71.7% of the variance in mean arousal in the linear model, *F*(1, 1347) = 3420.98, *p* < 0.001, again indicating higher mean valence was associated with lower mean arousal. A marginally non-significant quadratic regression model was found, *R*^*2*^ = 0.01, *p* = 0.07.

**Fig. 3:**
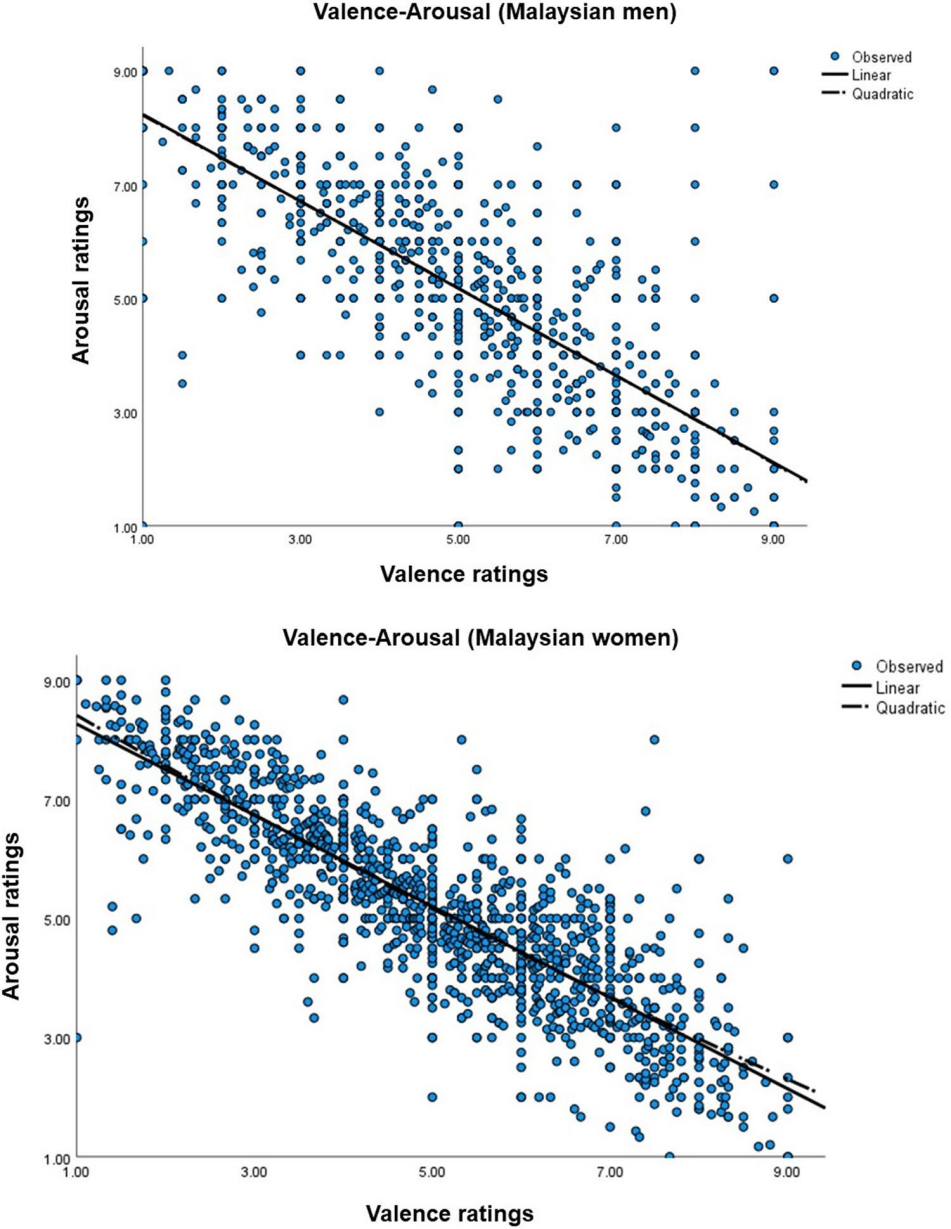
Quadratic function fitting arousal to the whole range of valence, for ratings of NAPS pictures by the Malaysian men (upper) and Malaysian women (lower)

Overall, the linear model provided the best fit for the Malaysian sample and suggested that higher levels of valence are associated with lower arousal. Both linear relationships deviate from the classical ‘boomerang’ shape found in the literature [52, 62]. See Fig. 3.

## Discussion

The aim of this study was to assess the suitability of for the Nencki Affective Picture System (NAPS) images for Malaysian participants and compare the results with the original European norms. It was hypothesised that cultural and gender differences between the Malaysian and European samples would be found in affective image ratings. As predicted, significant cultural differences were found in valence, arousal and motivation, Malaysians rated images significantly lower on valence and approach/avoidance (motivation), but higher on arousal than the European sample. However, there were no gender differences in any of the ratings. Notably, there was a linear relationship between valence and arousal in the Malaysian sample as compared to the classic ‘boomerang’ shape found in previous studies [52, 62].

### Cross-cultural and gender comparisons

Overall, Malaysian participants provided lower valence ratings compared to the Europeans. This pattern aligns with research showing that Malaysians, influenced by collectivistic cultural values, typically exhibit greater emotional suppression compared to their individualistic European counterparts [90]. Supporting evidence has also suggested that Malaysians demonstrate distinct patterns of emotional processing, including more nuanced recognition of specific emotions [91] and different emotional engagement with cultural stimuli [92], highlighting how cultural context might shape emotional experiences and expressions. It may also be that European cultures emphasize individual emotional expression, fostering higher valence ratings; on the other hand, Malaysians who live in a collectivistic society may be more inclined towards emotional moderation, affecting valence perceptions. However, future research should ascertain this claim more closely.

Malaysian participants reported higher arousal levels than European participants, regardless of gender, consistent with Rahman and Reza’s study [77] in which Malaysian participants provided higher arousal ratings than the original US sample. This might be explained by cultural differences in emotional regulation strategies, with Malaysians exhibiting more interpersonal emotion regulation, which may contribute to heightened arousal [90]. In Jobson’s study, Malaysians reported higher levels of fatalism and cultural beliefs about adversity than Australian participants, which could heighten emotional arousal, as individuals from collectivistic cultures tend to experience emotions more intensely due to strong family and community ties.

Similarly, to valence, the original European sample [52] provided higher ratings for approach/avoidance than the Malaysian participants. This finding is supported by previous research that Malaysian participants tend to practice restraint and non-confrontational approaches in a game on ultimatums, consequently leading to lower approach or higher avoidance tendencies than British participants [93]. In line with collectivistic cultural norms, the Malaysian participants in Chuah et al.’s study exhibited more cautious, non-confrontational behaviours compared to British participants who demonstrated more assertive and higher approach tendencies. However, there remains a dearth of studies to explain this finding; therefore, future studies should be conducted to explore these mechanisms further.

While previous studies found gender differences in approach/avoidance ratings, this was no longer the case after Bonferroni correction. For instance, males tend to show greater approach bias towards cues predicting conflict in behavioural tasks, while females demonstrate more cautious exploration behaviours, leading to lower navigation performance [94, 95]. Additionally, studies on sexual stimuli reveal that automatic approach-avoidance tendencies are more pronounced in males, suggesting a stronger inclination towards approach behaviours [96]. In contrast, females often display higher avoidance tendencies, as evidenced by their greater avoidance and repetitive negative thinking in anxiety-provoking situations [97]. This pattern highlights a significant gender difference in approach-avoidance dynamics, with males typically favouring approach behaviours over avoidance.

Taken together, the hypothesis that significant cultural differences exist is therefore supported, in line with previous validation studies [56, 62, 98]. While gender differences were found in valence, arousal and approach/avoidance ratings in the European sample, there were no gender differences in the Malaysian sample for valence, arousal, and motivation ratings. In summary, while cultural differences were evident between Malaysians and Europeans, no notable gender differences emerged within the Malaysian sample.

### Valence and arousal affective space

Although gender comparisons were non-significant in the Malaysian sample, minor differences were revealed in the relationships between valence and arousal. While the valence-arousal relationship for Malaysian men was strictly linear, the quadratic regression for Malaysian women revealed a less linear relationship (*p* = 0.07). Both samples deviate from the classical ‘boomerang’ shape found in the literature [52], but are consistent with the linear relationship found among Chinese older adults [56].

The linearity of the valence-arousal relationship found in the Malaysian sample provides some support for the role of emotional suppression as a cultural adaptation. The strong negative association, with higher valence associated with lower arousal, could reflect a tendency for Malaysians to suppress expressed arousal even when experiencing pleasant emotions. If Malaysians felt comfortable freely expressing emotions across contexts, a “flatter” quadratic relationship might emerge, with those feeling moderately pleasant exhibiting similar arousal to those reporting extreme positive valence. Instead, the linear relationship is in line with broader cultural norms which support the notion that Malaysians may integrate emotional suppression as a useful strategy to conform to social norms [99]; however, more research should further ascertain this claim. To our knowledge, only one study has found a linear relationship in the valence and arousal ratings by Chinese older adults, another culture that values social harmony and suppressed outward emotional expression—on the IAPS [56], thereby supporting the current findings.

Taken together, the lower valence and motivation ratings found in the Malaysian sample compared to the European sample suggests that Malaysians may suppress outward displays of emotions, even when rating their reactions privately in an anonymous survey. Indeed, emotional restraint is encouraged to maintain social harmony across various Asian cultures [99–102]; these strategies for successful social interactions may be internalised, even in anonymous or non-social contexts. Expressing high positive affect towards stimuli, whether positive or negative, could disrupt social harmony while restraint enables smooth social functioning [103]. The ability to contain and regulate emotional dissatisfaction is seen as a crucial element of social etiquette in Malaysia [99].

### Strengths and implications

To our knowledge, this study is the first to provide complete NAPS affective ratings for a Malaysian sample, as well as direct comparisons between Malaysian and Western ratings, thereby improving our understanding of cultural differences in affective ratings. While previous studies with Malaysian samples have used a subset of the IAPS [77] and a separate set of emotional images [16], neither have validated nor provided normative ratings for a complete image set. Although a subset of the IAPS has been validated in a Malaysian sample [77], complete NAPS norms in the current study would provide researchers with another alternative to explore how affective pictures may affect valence, arousal and motivation (approach/avoidance) among Malaysians. While more research is needed to ascertain if these norms could also apply to diverse Southeast Asian samples due to possible cross-cultural nuances in emotional expression and communication styles, the significant differences in valence, arousal and approach/avoidance ratings between the Malaysian and European samples highlight the necessity for more cross-cultural validations.

The current findings underscore the importance of recognising cultural nuances in affective image stimuli, as it is apparent in the literature that Western and Eastern cultures respond differently to images, indicating Western standardisation data often does not apply to Asian contexts [56, 104]. However, it is important to avoid simplified dichotomy of West vs East as there are findings that show inconsistent patterns in responding to affective images, e.g. research done on an Indian [61] and Iranian samples [62].

The validated affective image set has potential practical implications, particularly as a non-invasive and cost-effective screening tool for mood disorders in Southeast Asian populations. By addressing cultural nuances in symptom expression, this tool could enhance the accuracy of mental health assessments in these contexts. Future studies may compare responses from clinical populations against the validated indices, further refining our understanding of mood disorders within this cultural framework. Importantly, this approach could offer an alternative to traditional face-to-face assessments, which often face limitations such as geographical barriers, stigma associated with mental health consultations, and other access-related obstacles [105]. As a standardized and easily implementable tool, it has the potential for wide-scale application across various healthcare settings, potentially leading to earlier identification of mood disorders.

### Limitations and future directions

There are a few limitations in this study to be mentioned. While results from the current study may suggest restraint in emotional expression, it is also possible that Malaysian participants may have felt strong valence, arousal and approach/avoidance tendencies but did not rate extreme ends of the rating scale, as demonstrated in previous studies on Asian participants [106].

Given the study's online format, participants were not given a time limit to evaluate the images, as the loading time for each image may be partially affected by participants’ internet connection. For this initial validation study, it was crucial to ensure that all 1356 image ratings were applicable to a Malaysian sample. Additionally, this validation process allows the research team and other researchers to examine which images elicited the most extreme ratings in terms of valence, arousal, and approach/avoidance tendencies. This information will enable future research to utilise these images in controlled laboratory environments, including precise timing for each trial.

Physiological measures such as galvanic skin response (GSR), heart rate and pupil dilation would also provide further clarity into these findings in future in-lab studies; however, this was not feasible for online data collection in the current study. As images were presented randomly to each participant, the number of evaluations per image was not equal, which may have impacted the ratings to some extent.

Future studies may also consider the role of personality with participants’ reactivity to affective images. Some studies have suggested that personality traits may influence the relationship between valence and arousal, resulting a linear rather than a classic ‘boomerang’-shaped relationship. While higher levels of extraversion have been associated with increased arousal and positive valence [107], higher levels of neuroticism (sometimes known as negative emotionality) have been associated with increased arousal with negative valence, which could flatten the expected boomerang-shape [108]. Openness to experience was associated with positive valence-low arousal IAPS pictures [109]. Thus, future studies may consider measuring affective ratings with personality correlates.

The current findings were based on a general adult Malaysian sample aged above 18 years old. Future studies may also examine if older and younger Malaysian adults demonstrate distinct patterns in VAM ratings, as it is plausible that maturity and life experience may have influenced ratings. This is supported by prior studies that found age-related differences in emotional processing among Chinese adults [56] and older Swiss adults [110]. Additionally, Teismann et al. [111] demonstrated that age-related changes in emotional processing can influence arousal ratings, and emotional states like anxiety and depression can alter the perception of valence and arousal.

## Conclusion

This study provides the first complete set of normative ratings for the NAPS image set from a Malaysian sample, which will be available on a publicly accessible repository. In contrast with the original European norms by Marchewka et al. [52], Malaysian participants provided lower valence and motivation (approach/avoidance) ratings, suggesting a tendency towards emotional restraint which is favoured in social interactions. Taken as a whole, these cultural differences have likely stemmed from Asian cultural norms which emphasis social harmony over emotional intensity.

## Supplementary Information

Below is the link to the electronic supplementary material.

Supplementary file 1

Supplementary file 2

Supplementary file 3

## Data Availability

The data file is publicly available on OSF: https://osf.io/3xm4t/files/osfstorage.

## References

[1] Barrett LF. Valence is a basic building block of emotional life. J Res Pers. 2006;40(1):35–55. 10.1016/j.jrp.2005.08.006.

[2] Raz G, Touroutoglou A, Wilson-Mendenhall C, Gilam G, Lin T, Gonen T, et al. Functional connectivity dynamics during film viewing reveal common networks for different emotional experiences. Cogn Affect Behav Neurosci. 2016;16(4):709–23. 10.3758/s13415-016-0425-4. 27142636 10.3758/s13415-016-0425-4

[3] Schutz PA, Benson J, Decuir-Gunby JT. Approach/avoidance motives, test emotions, and emotional regulation related to testing. Anxiety Stress Coping. 2008;21(3):263–81. 10.1080/10615800701787672. 18612854 10.1080/10615800701787672

[4] Watson D, Wiese D, Vaidya J, Tellegen A. The two general activation systems of affect: structural findings, evolutionary considerations, and psychobiological evidence. J Pers Soc Psychol. 1999;76(5):820–38. 10.1037/0022-3514.76.5.820.

[5] Skerry AE, Saxe R. Neural representations of emotion are organized around abstract event features. Curr Biol. 2015;25(15):1945–54. 10.1016/j.cub.2015.06.009. 26212878 10.1016/j.cub.2015.06.009 PMC4824044

[6] Russell JA, Barrett LF. Core affect, prototypical emotional episodes, and other things called emotion: dissecting the elephant. J Pers Soc Psychol. 1999;76(5):805–19. 10.1037/0022-3514.76.5.805. 10353204 10.1037//0022-3514.76.5.805

[7] Russell JA. A circumplex model of affect. J Pers Soc Psychol. 1980;39(6):1161–78. 10.1037/h0077714.

[8] Barrett LF, Russell JA. The structure of current affect: controversies and emerging consensus. Curr Dir Psychol Sci. 1999;8(1):10–4. 10.1111/1467-8721.00003.

[9] Russell JA. Core affect and the psychological construction of emotion. Psychol Rev. 2003;110(1):145–72. 10.1037/0033-295X.110.1.145. 12529060 10.1037/0033-295x.110.1.145

[10] Kragel PA, LaBar KS. Multivariate neural biomarkers of emotional states are categorically distinct. Soc Cogn Affect Neurosci. 2015;10(11):1437–48. 10.1093/scan/nsv032. 25813790 10.1093/scan/nsv032 PMC4631142

[11] Weidman AC, Steckler CM, Tracy JL. The jingle and jangle of emotion assessment: imprecise measurement, casual scale usage, and conceptual fuzziness in emotion research. Emotion. 2017;17(2):267–95. 10.1037/emo0000226. 27642656 10.1037/emo0000226

[12] Chikazoe J, Lee DH, Kriegeskorte N, Anderson AK. Population coding of affect across stimuli, modalities and individuals. Nat Neurosci. 2014;17(8):1114–22. 10.1038/nn.3749. 24952643 10.1038/nn.3749 PMC4317366

[13] Campbell NM, Dawel A, Edwards M, Goodhew SC. Motivational direction diverges from valence for sadness, anger, and amusement: a role for appraisals? Emotion. 2023;23(5):1334–48. 10.1037/emo0001165. 36074620 10.1037/emo0001165

[14] Klein RJ, Jacobson NC, Robinson MD. A psychological flexibility perspective on well-being: emotional reactivity, adaptive choices, and daily experiences. Emotion. 2023;23(4):911–24. 10.1037/emo0001159. 36048033 10.1037/emo0001159 PMC10035040

[15] Long F, Ye C, Li Z, Tian Y, Liu Q. Negative emotional state modulates visual working memory in the late consolidation phase. Cogn Emot. 2020;34(8):1646–63. 10.1080/02699931.2020.1795626. 32686579 10.1080/02699931.2020.1795626

[16] Nasser N, Sharifat H, Abdul Rashid A, Ab Hamid S, Abdul Rahim E, Mohamad M, et al. Validation of emotional stimuli flashcards for conducting ‘response to reward’ fMRI study among Malaysian undergraduates. JSM. 2020;49(11):2773–83. 10.1757/jsm-2020-4911-16.

[17] Cuétara I. Emotional development in people with high capacities: induction of emotions through pictorial abstraction. Sustainability. 2020;23(12):5912. 10.3390/su12155912.

[18] Jiang M, Hassan A, Chen Q, Liu Y. Effects of different landscape visual stimuli on psychophysiological responses in Chinese students. Indoor Built Environ. 2020;29(7):1006–16. 10.1177/1420326X19870578.

[19] Witten E, Ryynanen J, Wisdom S, Tipp C, Chan SWY. Effects of soothing images and soothing sounds on mood and well-being. Br J Clin Psychol. 2023;62(1):158–79. 10.1111/bjc.12400. 36342851 10.1111/bjc.12400

[20] Zempelin S, Sejunaite K, Lanza C, Riepe MW. Emotion induction in young and old persons on watching movie segments: facial expressions reflect subjective ratings. PLoS ONE. 2021;16(6):e0253378. 10.1371/journal.pone.0253378. 34143827 10.1371/journal.pone.0253378 PMC8213152

[21] Devilly G, O’Donohue R. A video is worth a thousand thoughts: comparing a video mood induction procedure to an autobiographical recall technique. Aust J Psychol. 2021;15(73):1–14. 10.1080/00049530.2021.1997553.

[22] Meuwese D, Dijkstra K, Maas J, Koole SL. Beating the blues by viewing green: depressive symptoms predict greater restoration from stress and negative affect after viewing a nature video. J Environ Psychol. 2021;1(75): 101594. 10.1016/j.jenvp.2021.101594.

[23] Bodenschatz CM, Czepluch F, Kersting A, Suslow T. Efficient visual search for facial emotions in patients with major depression. BMC Psychiatry. 2021;21(1):92. 10.1186/s12888-021-03093-6. 33573637 10.1186/s12888-021-03093-6 PMC7879523

[24] Song XM, Hu XW, Li Z, Gao Y, Ju X, Liu DY, et al. Reduction of higher-order occipital GABA and impaired visual perception in acute major depressive disorder. Mol Psychiatry. 2021;26(11):6747–55. 10.1038/s41380-021-01090-5. 33863994 10.1038/s41380-021-01090-5 PMC8760062

[25] Dal Fabbro D, Catissi G, Borba G, Lima L, Hingst-Zaher E, Rosa J, et al. e-nature positive emotions photography database (e-NatPOEM): affectively rated nature images promoting positive emotions. Sci Rep. 2021;11(1):11696. 10.1038/s41598-021-91013-9. 34083616 10.1038/s41598-021-91013-9 PMC8175760

[26] Balsamo M, Carlucci L, Padulo C, Perfetti B, Fairfield B. A bottom-up validation of the IAPS, GAPED, and NAPS affective picture databases: differential effects on behavioral performance. Front Psychol. 2020;4:11. 10.3389/fpsyg.2020.02187. 10.3389/fpsyg.2020.02187 PMC7498678 33013565

[27] Zamora E, Richards M, Introzzi I, Aydmune Y, Urquijo S, Guàrdia J, et al. The Nencki affective picture system (NAPS): a children-rated subset. Trends Psycho. 2020. 10.1007/s43076-020-00029-z.

[28] Out C, Goudbeek M, Krahmer E. Gradual positive and negative affect induction: the effect of verbalizing affective content. PLoS ONE. 2020;15(5):e0233592. 10.1371/journal.pone.0233592. 32469910 10.1371/journal.pone.0233592 PMC7259663

[29] Zimmermann P, Guttormsen S, Danuser B, Gomez P. Affective computing—a rationale for measuring mood with mouse and keyboard. Int J Occup Saf Ergon. 2003;9(4):539–51. 10.1080/10803548.2003.11076589. 14675525 10.1080/10803548.2003.11076589

[30] Wilhelm P, Schoebi D. Assessing mood in daily life. Eur J Psychol Assess. 2007;23(4):258–67. 10.1027/1015-5759.23.4.258.

[31] Scherer KR. What are emotions? And how can they be measured? Soc Sci Inf. 2005;44:695–729. 10.1177/05390184050582.

[32] Ruby FJM, Smallwood J, Engen H, Singer T. How self-generated thought shapes mood—the relation between mind-wandering and mood depends on the socio-temporal content of thoughts. PLoS ONE. 2013;8(10):e77554. 10.1371/journal.pone.0077554. 24194889 10.1371/journal.pone.0077554 PMC3806791

[33] Hume, D. (2012). Emotions and moods. Organizational behavior, (258–297).

[34] Mitchell RLC, Phillips LH. The psychological, neurochemical and functional neuroanatomical mediators of the effects of positive and negative mood on executive functions. Neuropsychologia. 2007;45(4):617–29. 10.1016/j.neuropsychologia.2006.06.030. 16962146 10.1016/j.neuropsychologia.2006.06.030

[35] Bogie BJ, Persaud MR, Smith D, Kapczinski FP, Frey BN. Explicit emotional memory biases in mood disorders: a systematic review. Psychiatry Res. 2019;1(278):162–72. 10.1016/j.psychres.2019.06.003. 10.1016/j.psychres.2019.06.003 31200195

[36] Kavanagh DJ, Bower GH. Mood and self-efficacy: impact of joy and sadness on perceived capabilities. Cogn Ther Res. 1985;9(5):507–25.

[37] Schwarz N. Emotion, cognition, and decision making. Cogn Emot. 2000;14:433–40. 10.1080/026999300402745.

[38] De Rubeis RJ, Strunk DR, editors. The Oxford handbook of mood disorders. Oxford: Oxford University Press; 2017.

[39] Davis E, Greenberger E, Charles S, Chen C, Zhao L, Dong Q. Emotion experience and regulation in China and the United States: how do culture and gender shape emotion responding? Int J Psychol. 2012;47(3):230–9. 22250807 10.1080/00207594.2011.626043

[40] Gross JJ, Uusberg H, Uusberg A. Mental illness and well-being: an affect regulation perspective. World Psychiatry. 2019;18(2):130–9. 31059626 10.1002/wps.20618 PMC6502417

[41] Walker H, Kavedžija I. Values of happiness. HAU: J Ethnogr Theory. 2015;5(3):1–23. 10.1431/hau5.3.002.

[42] Elliott R, Zahn R, Deakin JF, Anderson IM. Affective cognition and its disruption in mood disorders. Neuropsychopharmacology. 2011;36(1):153–82. 10.1038/npp.2010.77. 20571485 10.1038/npp.2010.77 PMC3055516

[43] Campbell-Sills L, Barlow DH. Incorporating emotion regulation into conceptualizations and treatments of anxiety and mood disorders. In: Gross JJ, editor. Handbook of emotion regulation. New York: The Guilford Press; 2007. p. 542–59.

[44] Akram U, Drabble J, Cau G, Hershaw F, Rajenthran A, Lowe M, et al. Exploratory study on the role of emotion regulation in perceived valence, humour, and beneficial use of depressive internet memes in depression. Sci Rep. 2020;10(1):899. 10.1038/s41598-020-57953-4. 31965036 10.1038/s41598-020-57953-4 PMC6972852

[45] Linden DEJ, Habes I, Johnston SJ, Linden S, Tatineni R, Subramanian L, et al. Real-time self-regulation of emotion networks in patients with depression. PLoS ONE. 2012;7(6):e38115. 10.1371/journal.pone.0038115. 22675513 10.1371/journal.pone.0038115 PMC3366978

[46] Picó-Pérez M, Radua J, Steward T, Menchón JM, Soriano-Mas C. Emotion regulation in mood and anxiety disorders: a meta-analysis of fMRI cognitive reappraisal studies. Prog Neuropsychopharmacol Biol Psychiatry. 2017;3(79):96–104. 10.1016/j.pnpbp.2017.06.001. 10.1016/j.pnpbp.2017.06.001 28579400

[47] Lang PJ, Bradley MM, Cuthbert BN. International affective picture system (IAPS): affective ratings of pictures and instruction manual. Gainesville: University of Florida; 2008.

[48] Dan-Glauser ES, Scherer KR. The Geneva affective picture database (GAPED): a new 730-picture database focusing on valence and normative significance. Behav Res Methods. 2011;43:468–77. 21431997 10.3758/s13428-011-0064-1

[49] Kurdi B, Lozano S, Banaji MR. Introducing the open affective standardized image set (OASIS). Behav Res Methods. 2017;49:457–70. 26907748 10.3758/s13428-016-0715-3

[50] Moyal N, Henik A, Anholt GE. Categorized affective pictures database (CAP-D). J Cogn. 2018. 10.5334/joc.47. 31517214 10.5334/joc.47 PMC6634429

[51] Haberkamp A, Glombiewski JA, Schmidt F, Barke A. The DIsgust-RelaTed-Images (DIRTI) database: validation of a novel standardized set of disgust pictures. Behav Res Ther. 2017;1(89):86–94. 10.1016/j.brat.2016.11.010. 10.1016/j.brat.2016.11.010 27914317

[52] Marchewka A, Żurawski Ł, Jednoróg K, Grabowska A. The Nencki affective picture system (NAPS): Introduction to a novel, standardized, wide-range, high-quality, realistic picture database. Behav Res. 2014;46(2):596–610. 10.3758/s13428-013-0379-1. 10.3758/s13428-013-0379-1 PMC4030128 23996831

[53] Riegel M, Żurawski Ł, Wierzba M, Moslehi A, Klocek Ł, Horvat M, et al. Characterization of the Nencki affective picture system by discrete emotional categories (NAPS BE). Behav Res. 2016;48(2):600–12. 10.3758/s13428-015-0620-1. 10.3758/s13428-015-0620-1 PMC4891391 26205422

[54] Cordaro DT, Sun R, Keltner D, Kamble S, Huddar N, McNeil G. Universals and cultural variations in 22 emotional expressions across five cultures. Emotion. 2018;18(1):75–93. 10.1037/emo0000302. 28604039 10.1037/emo0000302

[55] Matsumoto D, Yoo SH, Nakagawa S. Culture, emotion regulation, and adjustment. J Pers Soc Psychol. 2008;94(6):925–37. 10.1037/0022-3514.94.6.925. 18505309 10.1037/0022-3514.94.6.925

[56] Gong X, Wang D. Applicability of the international affective picture system in Chinese older adults: a validation study. PsyCh J. 2016;5(2):117–24. 10.1002/pchj.131. 27256203 10.1002/pchj.131

[57] Shirai R, Watanabe K. Open biological negative image set. Royal Soc Open Sci. 2022;9(1):211128. 10.1098/rsos.211128. 10.1098/rsos.211128 PMC8728170 35070342

[58] Mavrou I, Dewaele J-M. Emotionality and pleasantness of mixed‐emotion stimuli: The role of language, modality, and emotional intelligence. Int J Appl Linguist. 2020;30(2):313–28.

[59] Huang J, Xu D, Peterson BS, Hu J, Cao L, Wei N, et al. Affective reactions differ between Chinese and American healthy young adults: a cross-cultural study using the international affective picture system. BMC Psychiatry. 2015;15(1):60. 10.1186/s12888-015-0442-9. 25885052 10.1186/s12888-015-0442-9 PMC4378560

[60] Mavrou I, Dewaele JM. Emotionality and pleasantness of mixed-emotion stimuli: the role of language, modality, and emotional intelligence. Int J Appl Linguist. 2020;30(2):313–28. 10.1111/ijal.12285.

[61] Lohani M, Gupta R, Srinivasan N. Cross-cultural evaluation of the international affective picture system on an Indian sample. Psychol Stud. 2013;58(3):233–41. 10.1007/s12646-013-0196-8.

[62] Riegel M, Moslehi A, Michałowski JM, Żurawski Ł, Horvat M, Wypych M, et al. Nencki affective picture system: cross-cultural study in Europe and Iran. Front Psychol. 2017;3:8. 10.3389/fpsyg.2017.00274. 10.3389/fpsyg.2017.00274 PMC5334317 28316576

[63] Compare Countries - Hofstede Insights. https://www.theculturefactor.com/country-comparison-tool

[64] Taras V, Rowney J, Steel P. Half a century of measuring culture: review of approaches, challenges, and limitations based on the analysis of 121 instruments for quantifying culture. J Int Manag. 2009;15(4):357–73. 10.1016/j.intman.2008.08.005.

[65] Krys K, Haas BW, Igou ER, Kosiarczyk A, Kocimska-Bortnowska A, Kwiatkowska A, et al. Introduction to a culturally sensitive measure of well-being: combining life satisfaction and interdependent happiness across 49 different cultures. J Happiness Stud. 2023;24(2):607–27. 10.1007/s10902-022-00588-1.

[66] Lim N. Cultural differences in emotion: differences in emotional arousal level between the East and the West. Integr Med Res. 2016;5(2):105–9. 10.1016/j.imr.2016.03.004. 28462104 10.1016/j.imr.2016.03.004 PMC5381435

[67] Reddy G, Gleibs IH. The endurance and contestations of colonial constructions of race among Malaysians and Singaporeans. Front Psychol. 2019;16:10. 10.3389/fpsyg.2019.00792. 10.3389/fpsyg.2019.00792 PMC6477069 31040805

[68] Changsong W. Cinema attendance and cinema-going audience in Malaysia. Media Watch. 2019;10(3):539–49. 10.1565/mw/2019/v10i3/49682.

[69] Lithari C, Frantzidis CA, Papadelis C, Vivas AB, Klados MA, Kourtidou-Papadeli C, et al. Are Females more responsive to emotional stimuli? A neurophysiological study across arousal and valence dimensions. Brain Topogr. 2010;23(1):27–40. 10.1007/s10548-009-0130-5. 20043199 10.1007/s10548-009-0130-5 PMC2816804

[70] Soares AP, Pinheiro AP, Costa A, Frade CS, Comesaña M, Pureza R. Adaptation of the international affective picture system (IAPS) for European Portuguese. Behav Res. 2015;47(4):1159–77. 10.3758/s13428-014-0535-2. 10.3758/s13428-014-0535-2 25381023

[71] Zhang W, Qiu L, Tang F, Sun HJ. Gender differences in cognitive and affective interpersonal emotion regulation in couples: an fNIRS hyperscanning. Soc Cogn Affect Neurosci. 2023;18(1):057. 10.3713/mjm.vol12.2.5.2023. 10.1093/scan/nsad057 PMC10612568 37837406

[72] Mikkelsen MB, Mehlsen M, O’Toole MS. Age-dependent reactivity to affective images: evidence for variation across emotion categories. Exp Aging Res. 2018;44(4):297–310. 10.1080/0361073X.2018.1477360. 29847218 10.1080/0361073X.2018.1477360

[73] Stevens EM, Frank D, Codispoti M, Kypriotakis G, Cinciripini PM, Claiborne K, et al. The late positive potentials evoked by cigarette-related and emotional images show no gender differences in smokers. Sci Rep. 2019;9(1):3240. 10.1038/s41598-019-39954-0. 30824792 10.1038/s41598-019-39954-0 PMC6397300

[74] Ascheid S, Wessa M, Linke JO. Effects of valence and arousal on implicit approach/ avoidance tendencies: a fMRI study. Neuropsychologia. 2019;1(131):333–41. 10.1016/j.neuropsychologia.2019.05.028. 10.1016/j.neuropsychologia.2019.05.028 31153965

[75] Kaczmarek LD, Behnke M, Enko J, Kosakowski M, Guzik P, Hughes BM. Splitting the affective atom: divergence of valence and approach-avoidance motivation during a dynamic emotional experience. Curr Psychol. 2021;40(7):3272–83. 10.1007/s12144-019-00264-3.

[76] Ruiz-Padial E, Pastor MC, Mercado F, et al. MATTER in emotion research: Spanish standardization of an affective image set. Behav Res. 2021;53:1973–85. 10.3758/s13428-021-01567-9. 10.3758/s13428-021-01567-9 33694080

[77] Rahman NA, Reza F. Rating of affective pictures of low and high arousal domain among Malaysian population. Int J Acad Res Bus Soc Sci. 2017;7(10):507–18.

[78] Faul F, Erdfelder E, Lang AG, Buchner A. G*Power 3: a flexible statistical power analysis program for the social, behavioral, and biomedical sciences. Behav Res Methods. 2007;39(2):175–91. 10.3758/BF03193146. 17695343 10.3758/bf03193146

[79] Finger H, Goeke C, Diekamp D, Standvoß K, König P. LabVanced: A Unified JavaScript Framework for Online Studies. 2017 International Conference on Computational Social Science IC2S2, July 10–13, 2016, Cologne, Germany

[80] Moeller B, Frings C. Remote binding counts: measuring distractor-response binding effects online. Psychol Res. 2021;85:2249–55. 10.1007/s00426-020-01413-1. 32894340 10.1007/s00426-020-01413-1 PMC8357652

[81] Obleser J, Kreitewolf J, Vielhauer R, et al. Circadian fluctuations in glucocorticoid level predict perceptual discrimination sensitivity. iScience. 2021;24:102345. 10.1371/journal.pone.0233592. 33870139 10.1016/j.isci.2021.102345 PMC8047178

[82] Sander I, Mazumder R, Fingerhut J, et al. Beyond built density: from coarse to fine-grained analyses of emotional experiences in urban environments. J Environ Psychol. 2024;96:102337. 10.1016/j.jenvp.2024.102337.

[83] Chládková K, Podlipský VJ, Nudga N, Šimáčková Š. The McGurk effect in the time of pandemic: age-dependent adaptation to an environmental loss of visual speech cues. Psychon Bull Rev. 2021;28:992–1002. 10.3758/s13423-020-01852-2. 33443708 10.3758/s13423-020-01852-2

[84] Starcke K, Mayr J, von Georgi R. Emotion modulation through music after sadness induction—the iso principle in a controlled experimental study. Int J Environ Res Public Health. 2021;18:12486. 34886210 10.3390/ijerph182312486 PMC8656869

[85] Watson D, Clark LA, Tellegen A. Development and validation of brief measures of positive and negative affect: the PANAS scales. J Pers Soc Psychol. 1988;54(6):1063–70. 10.1037/0022-3514.54.6.1063. 3397865 10.1037//0022-3514.54.6.1063

[86] Ali AM, Hori H, Kim Y, Kunugi H. The depression anxiety stress scale 8-items expresses robust psychometric properties as an ideal shorter version of the depression anxiety stress scale 21 among healthy respondents from three continents. Front Psychol. 2022;24:13. 10.3389/fpsyg.2022.799769. 10.3389/fpsyg.2022.799769 PMC9044488 35496141

[87] Ali AM, Alkhamees AA, Hallit S, Al-Dwaikat TN, Khatatbeh H, Al-Dossary SA. The Depression anxiety stress scale 8: investigating its cutoff scores in relevance to loneliness and burnout among dementia family caregivers. Sci Rep. 2024;14(1):13075. 10.1038/s41598-024-60127-1. 38844485 10.1038/s41598-024-60127-1 PMC11156668

[88] Haron H, Mustafa SMS, Alias RA. Gender Influences on Emotional Self-Regulation among Malaysian Academicians. International Journal of Innovation. 2010;1(1).

[89] Marof AA, Zheng D, Zaremohzzabieh Z. Gender differences in the function of music for emotion regulation development in everyday life: an experience sampling method study. Malays J Music. 2023;12(2):76–94.

[90] Jobson L, Qiu LS, Wong J, Li H, Lies J, Lau W, Bryant RA, Liddell BJ. Cultural differences in appraisals of control and posttraumatic stress disorder symptoms. Eur J Psychotraumatol. 2024;15(1):2358685. 10.3390/ijerph19031163. 38836340 10.1080/20008066.2024.2358685 PMC11155424

[91] Mohan SN, Mukhtar F, Jobson L. Protocol for a between-group experimental study examining cultural differences in emotion processing between Malay and Caucasian adults with and without major depressive disorder. BMJ Open. 2016;6(10):e012774. 10.1136/bmjopen-2016-012774. 27798019 10.1136/bmjopen-2016-012774 PMC5093675

[92] Yusoff N, Samsuri NA, Ayob S, Chang TY. Emotional expression of the Malaysian Chinese towards the Malay cultural heritage visualization. J Ethnic Cult Stud. 2019;6(3):53–63. 10.2933/ejecs/259.

[93] Chuah SH, Hoffmann R, Jones M, Williams G. Do cultures clash? Evidence from cross-national ultimatum game experiments. J Econ Behav Organ. 2007;64(1):35–48. 10.1016/j.jebo.2006.04.006.

[94] Gagnon KT, Cashdan EA, Stefanucci JK, Creem-Regehr SH. Sex differences in exploration behavior and the relationship to harm avoidance. Hum Nat. 2016;27:82–97. 26650605 10.1007/s12110-015-9248-1

[95] McNamara TA, Ito R. Relationship between voluntary ethanol drinking and approach-avoidance biases in the face of motivational conflict: novel sex-dependent associations in rats. Psychopharmacology. 2021;238:1817–32. 33783557 10.1007/s00213-021-05810-1

[96] Hofmann W, Friese M, Gschwendner T. Men on the “pull” automatic approach-avoidance tendencies and sexual interest behavior. Soc Psychol. 2009;40(2):73–8.

[97] Graham BM, Weiner S, Li SH. Gender differences in avoidance and repetitive negative thinking following symptom provocation in men and women with spider phobia. Br J Clin Psychol. 2020;59(4):565–77. 32955767 10.1111/bjc.12267

[98] Branco D, Goncalves OF, Badia SB. A systematic review of international affective picture system (IAPS) around the world. Sensors. 2023;23(8):3866. 10.3390/s23083866. 37112214 10.3390/s23083866 PMC10143386

[99] Abd Hadi NH, Midin M, Tong SF, Chan LF, Mohd Salleh Sahimi H, Ahmad Badayai AR, et al. Exploring Malaysian parents’ and teachers’ cultural conceptualization of adolescent social and emotional competencies: a qualitative formative study. Front Public Health. 2023. 10.3389/fpubh.2023.992863. 37033063 10.3389/fpubh.2023.992863 PMC10076560

[100] Kraus B, Kitayama S. Interdependent self-construal predicts emotion suppression in Asian Americans: an electro-cortical investigation. Biol Psychol. 2019;1(146):107733. 10.1016/j.biopsycho.2019.107733. 10.1016/j.biopsycho.2019.107733 31352031

[101] Murata A, Moser JS, Kitayama S. Culture shapes electrocortical responses during emotion suppression. Soc Cogn Affect Neurosci. 2013;8(5):595–601. 10.1093/scan/nss036. 22422803 10.1093/scan/nss036 PMC3682443

[102] Huwaë S, Schaafsma J. Cross-cultural differences in emotion suppression in everyday interactions. Int J Psychol. 2018;53(3):176–83. 10.1002/ijop.12283. 27168184 10.1002/ijop.12283

[103] Soto JA, Levenson RW, Ebling R. Cultures of moderation and expression: emotional experience, behavior, and physiology in Chinese Americans and Mexican Americans. Emotion. 2005;5(2):154–65. 15982081 10.1037/1528-3542.5.2.154

[104] Gomes N, Benrós MF, Semin GR. Validation of the open biological negative image set for a portuguese population: comparing Japanese and portuguese samples and an exploration of low-order visual properties of the stimuli. Behav Res. 2024;56(2):860–80. 10.3758/s13428-023-02090-9. 10.3758/s13428-023-02090-9 PMC10830772 36882667

[105] Ibrahim N, Amit N, Shahar S, Wee LH, Ismail R, Khairuddin R, Safien AM. Do depression literacy, mental illness beliefs and stigma influence mental health help-seeking attitude? A cross-sectional study of secondary school and university students from B40 households in Malaysia. BMC Public Health. 2019;19(4):1–8. 31196033 10.1186/s12889-019-6862-6 PMC6565530

[106] Mayer LA, Elliott MN, Haas A, Hays RD, Weinick RM. Less use of extreme response options by Asians to standardized care scenarios may explain some racial/ethnic differences in CAHPS scores. Med Care. 2016;54(1):38. 10.1097/MLR.0000000000000453. 26783857 10.1097/MLR.0000000000000453

[107] Kuppens P, Tuerlinckx F, Yik M, Koval P, Coosemans J, Zeng KJ, Russell JA. The relation between valence and arousal in subjective experience varies with personality and culture. J Pers. 2017;85(4):530–42. 27102867 10.1111/jopy.12258

[108] Kehoe EG, Toomey JM, Balsters JH, Bokde AL. Personality modulates the effects of emotional arousal and valence on brain activation. Soc Cogn Affect Neurosci. 2012;7(7):858–70. 21948954 10.1093/scan/nsr059 PMC3475359

[109] Tok S, Koyuncu M, Dural S, Catikkas F. Evaluation of international affective picture system (IAPS) ratings in an athlete population and its relations to personality. Personality Individ Differ. 2010;49(5):461–6. 10.1016/j.paid.2010.04.020.

[110] Grühn D, Scheibe S. Age-related differences in valence and arousal ratings of pictures from the international affective picture system (IAPS): do ratings become more extreme with age? Behav Res Methods. 2008;40(2):512–21. 10.3758/BRM.40.2.512. 18522062 10.3758/brm.40.2.512

[111] Teismann H, Kissler J, Berger K. Investigating the roles of age, sex, depression, and anxiety for valence and arousal ratings of words: a population-based study. BMC Psychol. 2020;8:118. 10.1186/s40359-020-00485-3. 33160414 10.1186/s40359-020-00485-3 PMC7648958

