# Supplementary figures and images for: Is a picture worth the same emotions everywhere? Validation of images from the Nencki affective picture system in Malaysia
Source: Discov Ment Health. 2024 Dec 2;4(1):61. doi: 10.1007/s44192-024-00116-y (PMC11612132; doi:10.1007/s44192-024-00116-y)

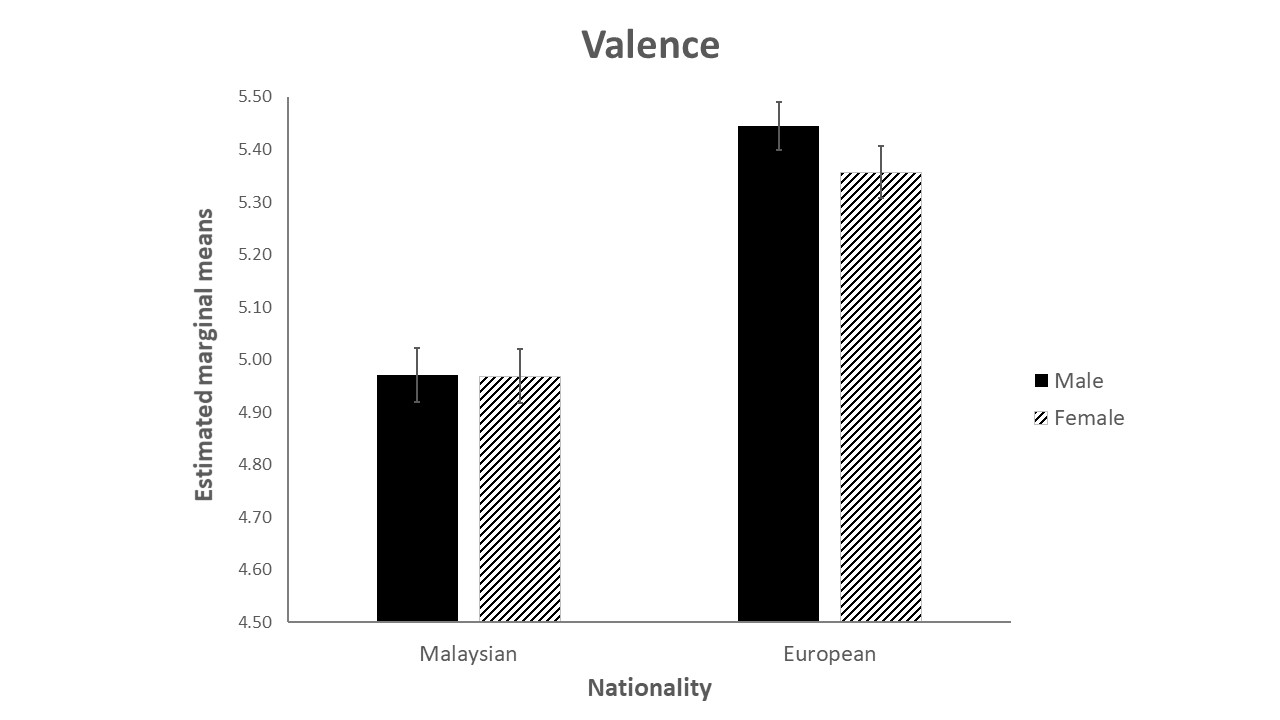

Supplement: Supplementary file 1 [file 44192_2024_116_MOESM1_ESM.jpg]

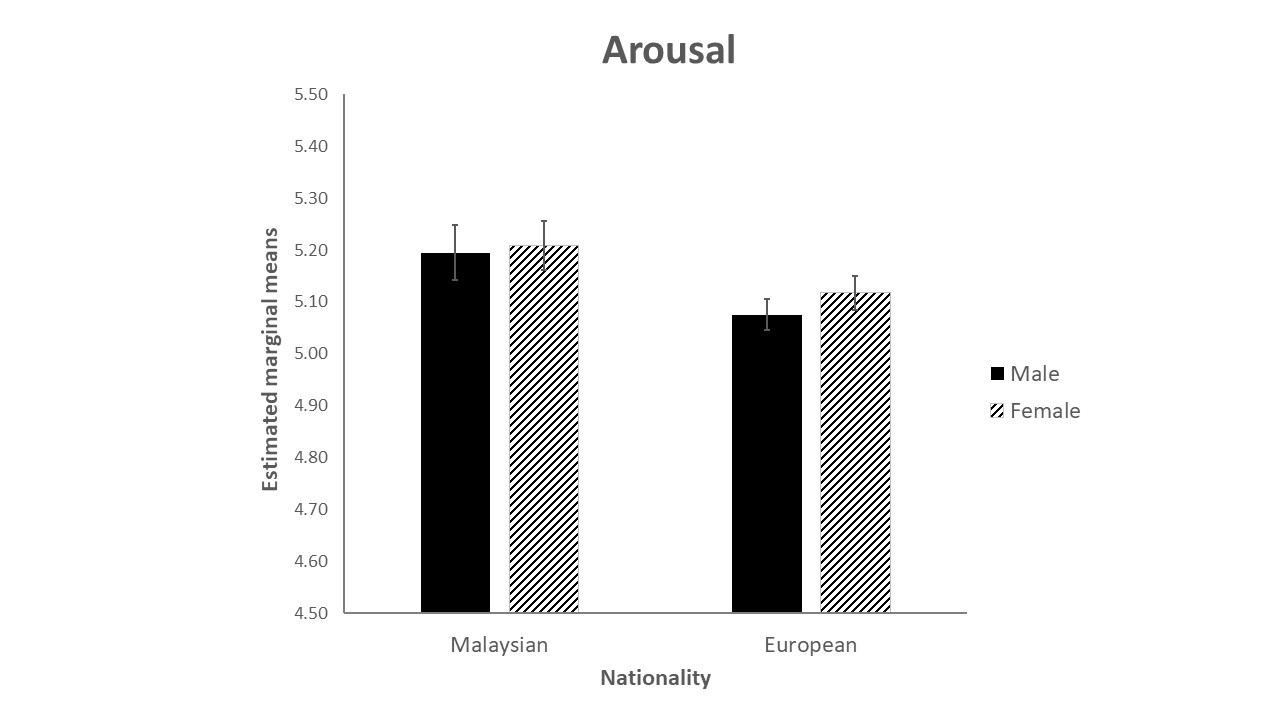

Supplement: Supplementary file 2 [file 44192_2024_116_MOESM2_ESM.jpg]

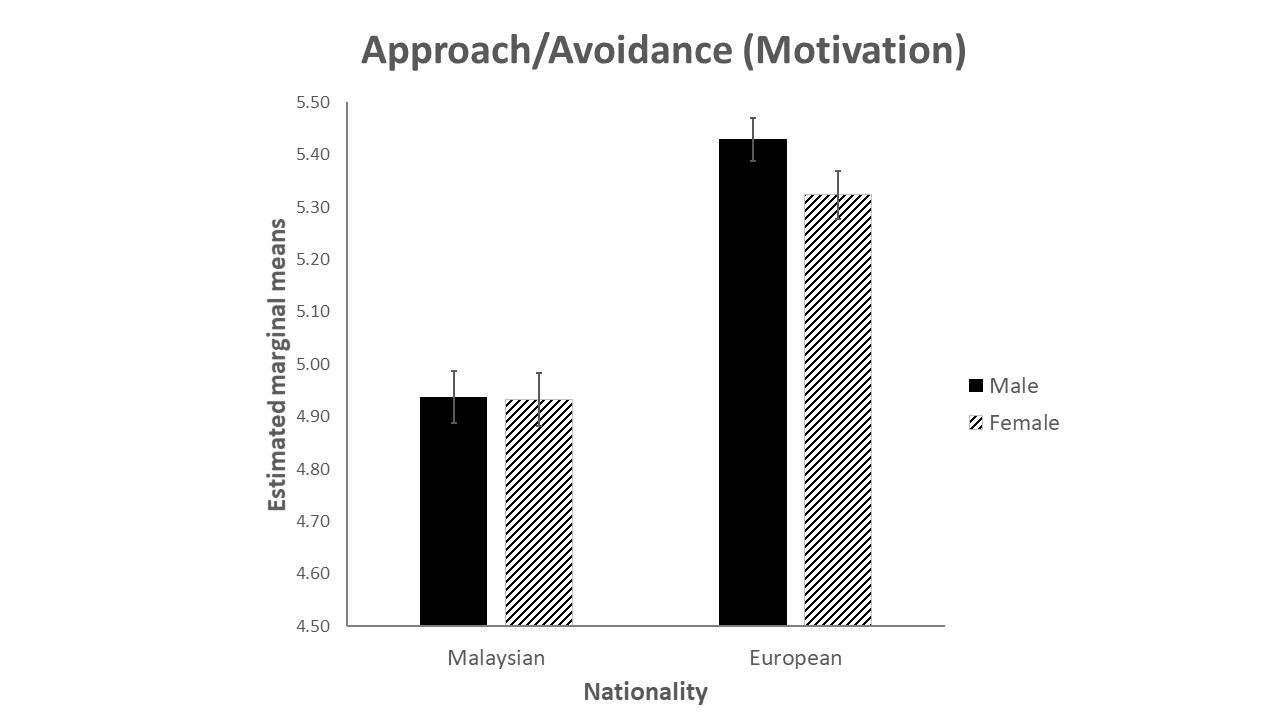

Supplement: Supplementary file 3 [file 44192_2024_116_MOESM3_ESM.jpg]
